# Optical Metabolic Imaging of Treatment Response in Human Head and Neck Squamous Cell Carcinoma

**DOI:** 10.1371/journal.pone.0090746

**Published:** 2014-03-04

**Authors:** Amy T. Shah, Michelle Demory Beckler, Alex J. Walsh, William P. Jones, Paula R. Pohlmann, Melissa C. Skala

**Affiliations:** 1 Department of Biomedical Engineering, Vanderbilt University, Nashville, Tennessee, United States of America; 2 Department of Medicine, Vanderbilt University Medical Center, Nashville, Tennessee, United States of America; 3 Department of Radiology, Vanderbilt University Medical Center, Nashville, Tennessee, United States of America; 4 Department of Medicine, Georgetown University Medical Center, Washington, District of Columbia, United States of America; Columbia University, United States of America

## Abstract

Optical metabolic imaging measures fluorescence intensity and lifetimes from metabolic cofactors nicotinamide adenine dinucleotide (NADH) and flavin adenine dinucleotide (FAD). These molecular level measurements provide unique biomarkers for early cellular responses to cancer treatments. Head and neck squamous cell carcinoma (HNSCC) is an attractive target for optical imaging because of easy access to the site using fiber optic probes. Two HNSCC cell lines, SCC25 and SCC61, were treated with Cetuximab (anti-EGFR antibody), BGT226 (PI3K/mTOR inhibitor), or cisplatin (chemotherapy) for 24 hours. Results show increased redox ratio, NADH α_1_ (contribution from free NADH), and FAD α_1_ (contribution from protein-bound FAD) for malignant cells compared with the nonmalignant cell line OKF6 (p<0.05). In SCC25 and SCC61 cells, the redox ratio is unaffected by cetuximab treatment and decreases with BGT226 and cisplatin treatment (p<0.05), and these results agree with standard measurements of proliferation rates after treatment. For SCC25, NADH α_1_ is reduced with BGT226 and cisplatin treatment. For SCC61, NADH α_1_ is reduced with cetuximab, BGT226, and cisplatin treatment. Trends in NADH α_1_ are statistically similar to changes in standard measurements of glycolytic rates after treatment. FAD α_1_ is reduced with cisplatin treatment (p<0.05). These shifts in optical endpoints reflect early metabolic changes induced by drug treatment. Overall, these results indicate that optical metabolic imaging has potential to detect early response to cancer treatment in HNSCC, enabling optimal treatment regimens and improved patient outcomes.

## Introduction

Head and neck squamous cell carcinoma (HNSCC) is the sixth leading cancer by incidence in the world [1]. Each year, 500,000 new cases are diagnosed with a five-year survival rate between 40–50% [1]. Current standards of care for HNSCC patients involves multidisciplinary care, including surgery, radiation therapy, chemotherapy, and rehabilitation. Treatment is intense since it is frequently delivered with curative aims. Resultant toxicities comprise nausea, vomiting, diarrhea, neuropathy, skin rash, dry mouth or thickened saliva, changes in taste, hypothyroidism, as well as impaired ability to speak, chew, and swallow [2] [3] [4]. These negative side effects from HNSCC treatment justify the need for improved treatments and the development of biomarkers of early treatment efficacy.

Current measures of treatment response in HNSCC include physical examination with endoscopy, x-ray computed tomography (CT), magnetic resonance imaging (MRI), and positron emission tomography (PET). Deep invasion of tumor and subtle changes to its dimensions during different treatment phases may not be measurable by physical exam. Imaging studies are only effective weeks to months after treatment begins and require contrast agents and/or expensive equipment. Therefore, these methods have low sensitivity to detect beneficial effects of treatment before several weeks have elapsed since treatment onset. Alternative treatment options for non-responders include re-irradiation, chemotherapy, or surgery [5]. Early predictors of drug efficacy would reduce toxicities, costs, and time associated with ineffective therapy. Therefore, there is a need for a cost-effective, noninvasive tool to determine treatment response at an early time point.

Therapeutic interventions for HNSCC include traditional chemotherapy and molecularly targeted inhibitors. Cisplatin is a common chemotherapy used in HNSCC [6]. In the past decade, targeted inhibitors have been developed to treat a number of solid tumors, including HNSCC. More than 90% of HNSCC cases exhibit upregulation of epidermal growth factor receptor (EGFR). The EGFR signaling pathway drives cell proliferation, growth, and survival. EGFR is the only proven molecular target for HNSCC therapy [5]. Cetuximab is a monoclonal antibody that effectively occludes ligand binding to EGFR, thereby inhibiting receptor activation, but clinical outcomes with cetuximab treatment vary and are not correlated with EGFR protein expression levels [7]. Therefore, downstream effectors, including phosphatidylinositol 3-kinase (PI3K) and mammalian target of rapamycin (mTOR), have been investigated as potential therapeutic targets. PI3K, a master regulator of metabolism, is mutated in about 37% of HNSCC [8]. BGT226 is a PI3K/mTOR inhibitor currently under clinical investigation for solid tumors [9]. However, there is a need for improved technologies to guide the selection of drugs for individual patients, so that alternative treatments such as BGT226 can be used at an early time point.

The EGFR and PI3K/mTOR signaling pathways regulate cellular metabolism, including glycolysis and oxidative phosphorylation [10]. Cancer often exhibits altered metabolism, particularly increased aerobic glycolysis (Warburg effect) [11]. During glycolysis, NAD^+^ is reduced to nicotinamide adenine dinucleotide (NADH). During oxidative phosphorylation, NADH is oxidized to NAD^+^ and FADH_2_ is oxidized to flavin adenine dinucleotide (FAD). NADH and FAD exhibit autofluorescence, whereas NAD^+^ and FADH_2_ do not. The optical redox ratio, defined as the fluorescence intensity of NADH divided by the fluorescence intensity of FAD, reflects relative amounts of glycolysis compared with oxidative phosphorylation and is an established method for probing cellular metabolism [12] [13] [14]. The fluorescence lifetime is the time a fluorophore stays in the excited state before relaxing to the ground state and reflects fluorophore microenvironment, including protein-binding and preferred metabolic pathways [15]. The optical redox ratio and fluorescence lifetimes of NADH and FAD exploit intrinsic contrast to measure optical endpoints of cellular metabolism. Furthermore, metabolic endpoints show particular promise because shifts in cellular metabolism often occur sooner than changes in tumor size or glucose uptake.

Tissue autofluorescence has been previously used to detect HNSCC. The autofluorescence intensity of NADH and FAD has been used to distinguish normal from dysplasia in oral tissue [16], and the NADH and FAD fluorescence lifetimes have been shown to identify precancer compared with normal in the DMBA-treated hamster cheek pouch model [17] [18] [19] [20]. Multiphoton microscopy of endogenous fluorescence has been used to quantify cellular and tissue morphology in the DMBA-treated hamster cheek pouch model [21] [22]. However, no previous literature has characterized endogenous fluorescence in response to treatment in HNSCC. Fluorescent dyes have been used to monitor anti-EGFR antibody uptake in HNSCC, but results did not reflect response *in vivo* [23] [24]. Optical metabolic imaging is sensitive to early metabolic shifts after cancer treatment and has potential to noninvasively detect treatment response sooner than current methods.

The serious morbidities and toxicities from HNSCC treatment, as well as treatment failures, justify the need for early predictors of treatment efficacy. This study tests the hypothesis that autofluorescence from metabolic cofactors NADH and FAD can resolve response to targeted therapies and chemotherapy in HNSCC. Optical metabolic imaging was performed on two HNSCC cell lines, SCC25 and SCC61, treated for 24 hours with targeted drugs (cetuximab or BGT226) or chemotherapy (cisplatin). HNSCC is an ideal site for optical imaging because of easy access to the site with fiber optic probes. These results indicate that optical metabolic imaging has potential to expedite drug screenings, develop optimal treatments, and improve patient outcomes for HNSCC.

## Materials and Methods

### Cell Culture and Reagents

The TERT-immortalized human oral keratinocyte line OKF6/TERT-1 (OKF6) [25], the squamous cell carcinoma line SCC25 [26] [27] [28] [29], and the squamous cell carcinoma line SCC61 [28] were acquired from J. Rheinwald and the Cell Culture Core of the Harvard Skin Disease Research Center, Boston, MA. OKF6 cells were cultured in keratinocyte serum-free medium (GIBCO K-sfm; Invitrogen) supplemented with 25 µg/ml bovine pituitary extract, 1% penicillin/streptomycin, 0.2 ng/ml epidermal growth factor, and 0.3 mM CaCl_2_. SCC25 and SCC61 cells were cultured in DMEM/F12 media (Invitrogen) supplemented with 10% fetal bovine serum and 0.4 µg/ml hydrocortisone (Sigma).

For fluorescence imaging, 10^5^ cells were plated on 35 mm glass-bottomed dishes (MatTek Corp.). The media was replaced 24 hours after plating with control media or treatment media containing 13 nM cetuximab (Vanderbilt Pharmacy), 300 nM NVP-BGT226 (Selleckchem), or 176 µM cisplatin (Selleckchem). The drug doses were chosen to be 11 times the IC50 for each drug [30] [31] [32]. The cells were imaged 24 hours after treatment.

### Imaging Instrumentation

Fluorescence lifetime images were collected using a custom-built multi-photon fluorescence microscope (Prairie Technologies). Images were acquired through an inverted microscope (TiE, Nikon) with a 40x oil immersion objective (1.3 NA). Fluorescence was excited using a titanium:sapphire laser (Chameleon, Coherent Inc.) and collected using a GaAsP photomultiplier tube (H7422P-40, Hamamatsu). NADH and FAD images were acquired sequentially for the same field of view. NADH fluorescence was isolated using an excitation wavelength of 750 nm and an emission bandpass filter of 400–480 nm. FAD fluorescence was isolated using an excitation wavelength of 890 nm and an emission bandpass filter of 500–600 nm. The average power incident on the sample was approximately 10 mW. The acquired images consisted of 256×256 pixels (170 µm×170 µm) with a 4.8 µs pixel dwell time. Time-correlated single photon counting (TCSPC) electronics (SPC-150, Becker and Hickl) were used to collect fluorescence lifetime images over 60 seconds. The approximate rate of photon counting was 1–2*10^5^ photons/second. The absence of photobleaching was confirmed by monitoring photon count rates throughout image acquisition.

The instrument response function (IRF) was measured from second harmonic generation of urea crystals excited at 900 nm, and the full width at half maximum (FWHM) was calculated to be 244 ps. A Fluoresbrite YG microsphere (Polysciences Inc.) was imaged as a daily standard. The lifetime decay curves were fit to a single exponential decay and the fluorescence lifetime was measured to be 2.13±0.28 ns (n = 7), which is consistent with published values [18] [33].

### Cyanide Perturbation

The OKF6 cells were plated at a density of 10^5^ cells per 35 mm glass-bottomed dish (MatTek Corp.). After 48 hours, fluorescence lifetime images of NADH and FAD were acquired. Then, the media was replaced with cyanide-supplemented media (4 mM NaCN, Sigma). After five minutes of cyanide treatment, fluorescence lifetime images of NADH and FAD were acquired.

### Image Analysis

Fluorescence lifetime images were analyzed using SPCImage software (Becker and Hickl). Binning included the selected pixel and the eight surrounding pixels. The fluorescence lifetimes were calculated by de-convolving the measured fluorescence decay curve with the IRF and fitting to a two-component exponential curve, F(t) = α_1_e^−t/τ1^+ α_2_e^−t/τ2^+ c. F(t) represents the fluorescence intensity as a function of time after the excitation pulse, τ_1_ and τ_2_ represent the short and long fluorescence lifetimes, respectively, α_1_ and α_2_ represent the contribution from each lifetime component (α_1_+α_2_ = 1), and c represents background light. A two-component decay curve was chosen to represent free and protein-bound conformations of NADH and FAD [18]. The weighted mean lifetime, τ_m_, was calculated, τ_m_ = α_1_τ_1_+α_2_τ_2_. The photon counts per pixel were summed over the 60 second collection time to calculate a fluorescence intensity image. A threshold was applied to exclude fluorescence from background and cell nuclei. The fluorescence intensities and lifetime values were imported into MATLAB (Mathworks) for further quantification. Redox ratio images were calculated by dividing the fluorescence intensity image of NADH by the fluorescence intensity image of FAD for the same field of view, and the average per image was computed. The redox ratio was normalized to control cells for comparing treatment groups within cell lines. The redox ratio was normalized to the nonmalignant OKF6 cells when comparing between cell lines. Average fluorescence lifetime values were calculated per image. For the control groups 30 images were analyzed, and for the treatment groups 18 images were analyzed.

### Western Blotting Analysis

Cells were plated at 3*10^6^ cells per 10 cm dish. After 24 hours, the media was removed, the cells were washed three times with phosphate buffered saline (PBS), and serum-free media was added. After another 24 hours, the media was replaced with treatment media for one hour. For the groups treated with epidermal growth factor (EGF) or transforming growth factor alpha (TGF-α), 10 ng/mL EGF or TGF-α was added for 5 minutes. The cells were lysed with lysis buffer (1% Triton X-100, 10% Glycerol, 50 mM HEPES pH 7.2, and 100 mM NaCl) supplemented with sodium orthovanadate and protease inhibitor cocktail. Proteins were separated using a 10% SDS-PAGE separation gel at 100 V. The gel was transferred at 27 V overnight to a PVDF membrane. The membrane was blocked using 5% bovine serum albumin (BSA) for one hour and then incubated in the following primary antibodies overnight: EGFR (Millipore, 1∶1000), pY1173 EGFR (Cell Signaling, 1∶250), AKT (Cell Signaling, 1∶250), pS473 AKT (Cell Signaling, 1∶250), or GAPDH (Sigma, 1∶1000). Membranes were washed four times, secondary antibodies were added for one hour, and electrogenerated chemiluminescence (ECL) was used to measure luminescence.

### Proliferation Assay

Cells were plated in a 96 well plate at 3.3*10^3^ cells per well. Four wells per treatment group were plated. After 24 hours, the media was replaced with treatment media supplemented with 10 µM BrdU. The cells incubated for 24 hours and then were fixed with 4% paraformaldehyde for 15 minutes. The cells were washed twice with PBS and permeabilized with 0.3% Triton X in PBS for 15 minutes. The DNA was denatured using hydrochloric acid, and the cells were blocked using 10% goat serum and 0.3% Triton X for one hour. Primary antibody (rat anti-BrdU, Abcam, 1∶100) was incubated overnight. The cells were washed three times with PBS and incubated in secondary antibody (DyLight594-conjugated goat anti-rat, Jackson Immunoresearch, 1∶200) for two hours. The cells were washed three times with PBS and counterstained with Hoechst 33528 (1∶1000) for 10 minutes. Fluorescence images were acquired for three fields of view per well (n = 12), and the number of cells per image was counted (ImageJ).

### Glucose and Lactate Assays

Cells were plated at a density of 10^5^ cells per 35 mm dish, and 24 hours later the media was replaced with treatment media. After 24 hours of treatment, glucose and lactate concentrations from four samples per group were measured according to the protocols of commercially available kits (Invitrogen; Eton Biosceience).

### Statistical Analyses

Bar graphs are represented as mean ± standard error. Statistical significance was determined using two-way Wilcoxon rank sum tests in MATLAB (Mathworks). A p-value less than 0.05 indicated statistical significance.

## Results

**Figure 1:**
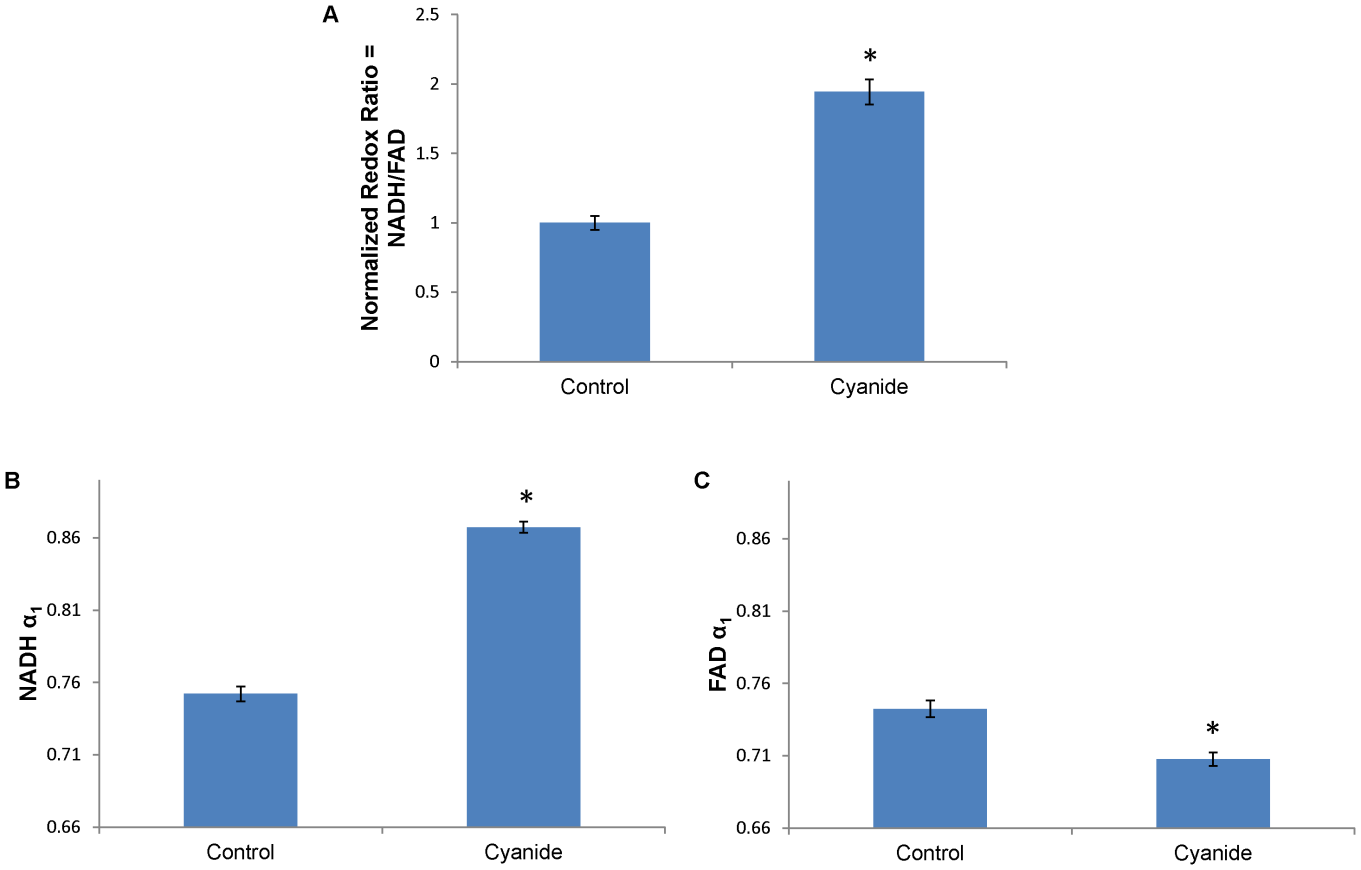
Cyanide treatment alters redox ratio, NADH α_1_, and FAD α_1_ in nonmalignant oral cells (OKF6). (a) Cyanide treatment (4 mM) disrupts the electron transport chain, causing an increase in the optical redox ratio. (b) Cyanide treatment increases the contribution of free NADH (α_1_) and (c) decreases the contribution of protein-bound FAD (α_1_). *p<0.05, rank sum test; mean ± SEM.

Redox ratio validation was performed by perturbing nonmalignant OKF6 cells with 4 mM cyanide, which prevents oxidation of NADH to NAD^+^ in the electron transport chain [34]. This accumulation of NADH causes an increase in the optical redox ratio (Figure 1a) and verifies isolation of NADH and FAD fluorescence. The contribution from free NADH (α_1_) increases with cyanide treatment (Figure 1b), causing a decreased NADH mean lifetime (data not shown). The contribution from protein-bound FAD (α_1_) decreases with cyanide treatment (Figure 1c), causing an increased FAD mean lifetime (data not shown).

**Figure 2:**
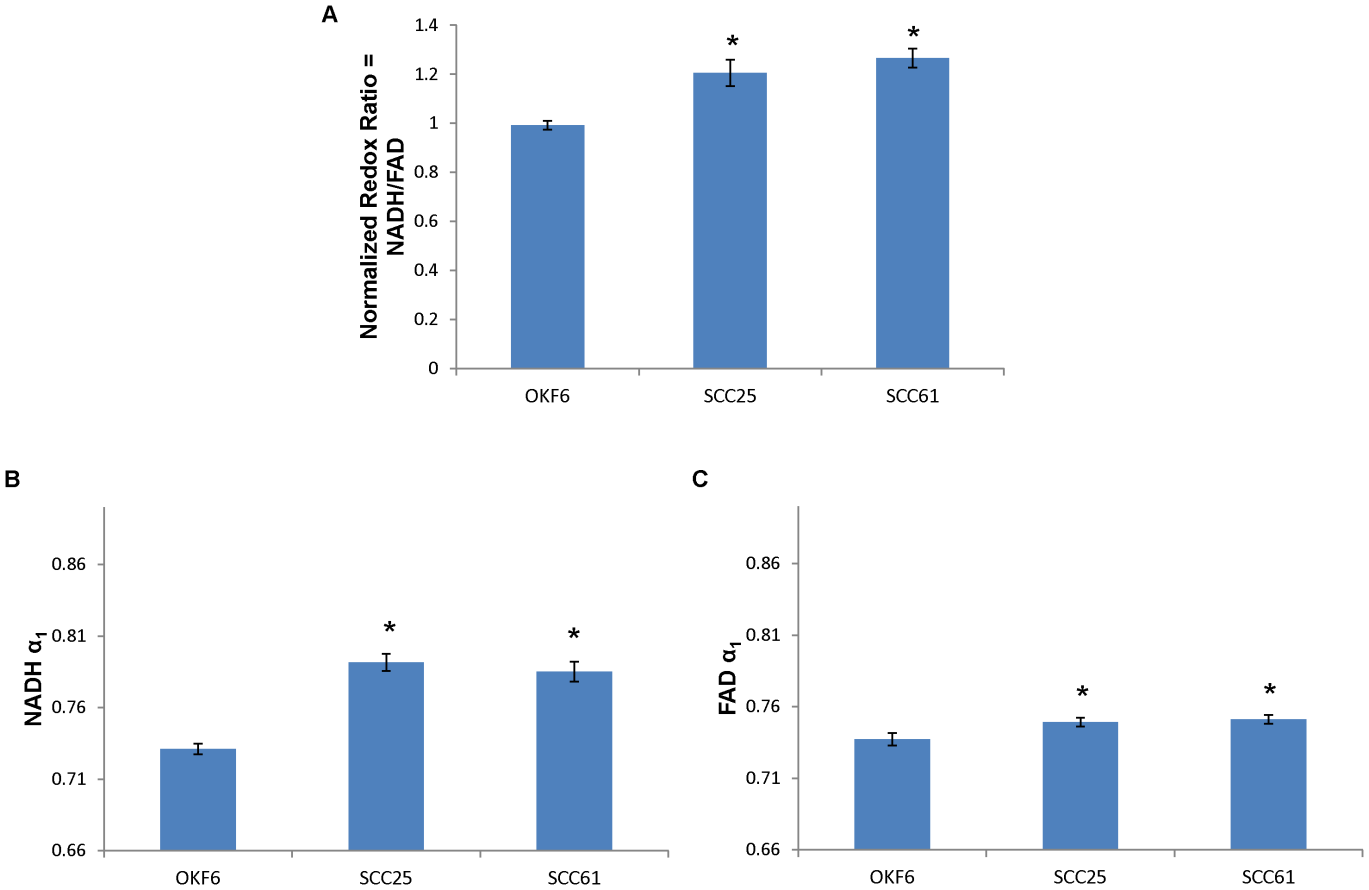
Optical metabolic endpoints distinguish malignant from nonmalignant cells. (a) The normalized redox ratio increases for the malignant cell lines (SCC25 and SCC61) compared to nonmalignant cells (OKF6), indicating increased glycolysis compared with oxidative phosphorylation. (b-c) The contribution of free NADH and protein-bound FAD (α_1_) increase for the malignant cell lines compared with the nonmalignant cell line, reflecting shifts in metabolic pathways. *p<0.05, rank sum test; mean ± SEM.

The optical metabolic endpoints differentiate the malignant cell lines, SCC25 and SCC61, from the nonmalignant cell line, OKF6 (Figure 2). The malignant cell lines showed an increased redox ratio compared with the OKF6 cells (p<0.05). The malignant cell lines showed increased NADH α_1_ compared with the OKF6 cells (p<0.05) and increased FAD α_1_ compared with the OKF6 cells (p<0.05), suggesting differences in metabolic pathways between the malignant and nonmalignant cells.

**Figure 3:**
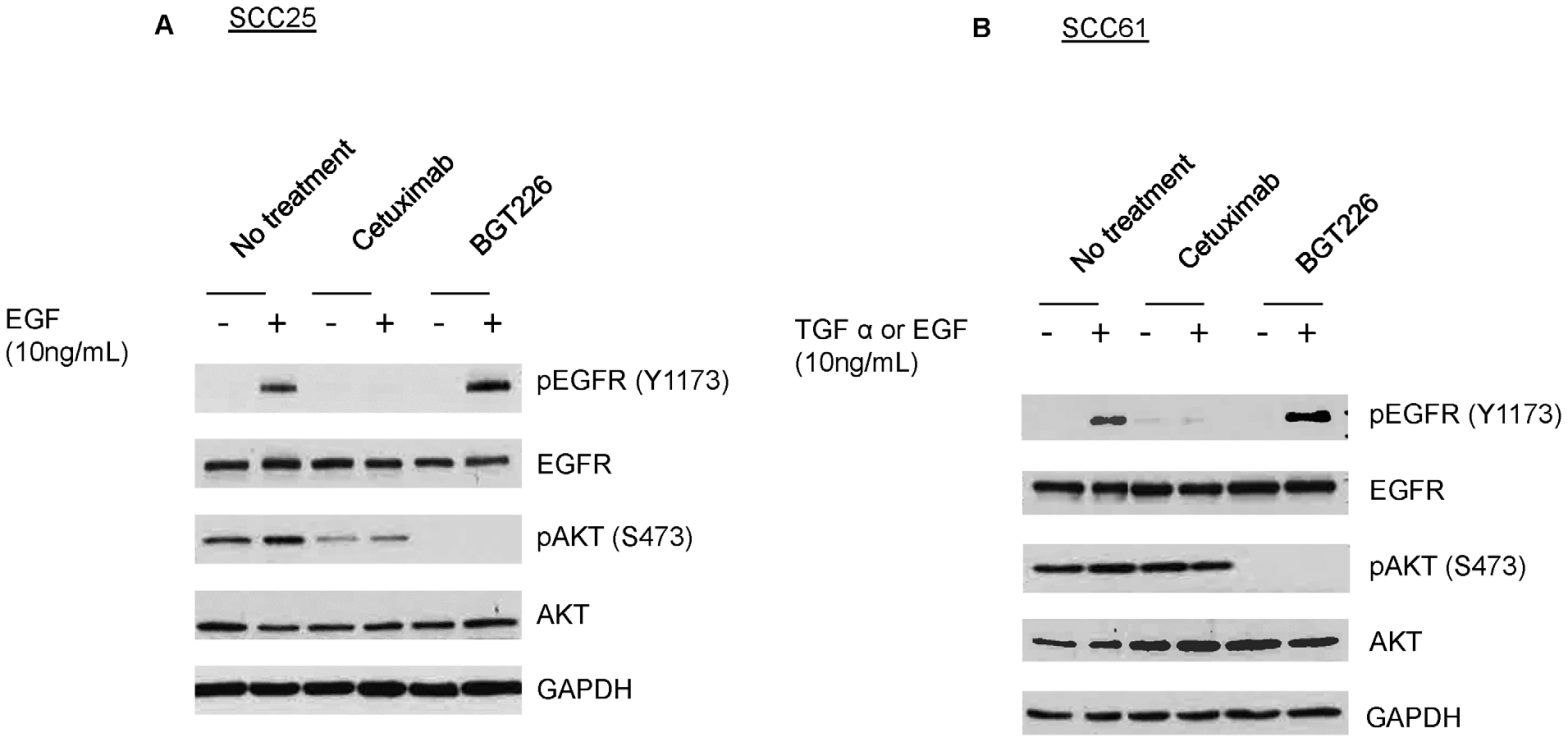
Western blotting analysis verifies molecular targeting of cetuximab and BGT226. Western blot for (a) SCC25 and (b) SCC61 cells. Epidermal growth factor (EGF) and transforming growth factor alpha (TGF α) activate the epidermal growth factor receptor (EGFR) and AKT pathways. Treatment with cetuximab decreases phosphorylated EGFR (pEGFR), and treatment with BGT226 decreases phosphorylated AKT (pAKT).

Western blotting analysis was used to ensure target inhibition (Figure 3). Cetuximab targeting of EGFR was assessed by measuring phosphorylated tyrosine (Y) 1173 of EGFR (pEGFR), which is absent with cetuximab treatment. BGT226 targeting of PI3K/mTOR was assessed by measuring phosphorylated serine (S) 473 of Akt (pAkt) because PI3K and mTOR activation drive Akt activation in the PI3K/Akt signaling pathway. pAkt is absent with BGT226 treatment. These results indicate that cetuximab and BGT226 target EGFR and PI3K/mTOR, respectively. Western blotting analysis was also performed to characterize the SCC25 and SCC61 cell lines (not shown). SCC61 cells showed increased pAkt, reflecting upregulated PI3K, and agreeing with published results [35]. Additionally, SCC61 cells exhibited increased EGFR and pEGFR compared with SCC25 cells.

**Figure 4:**
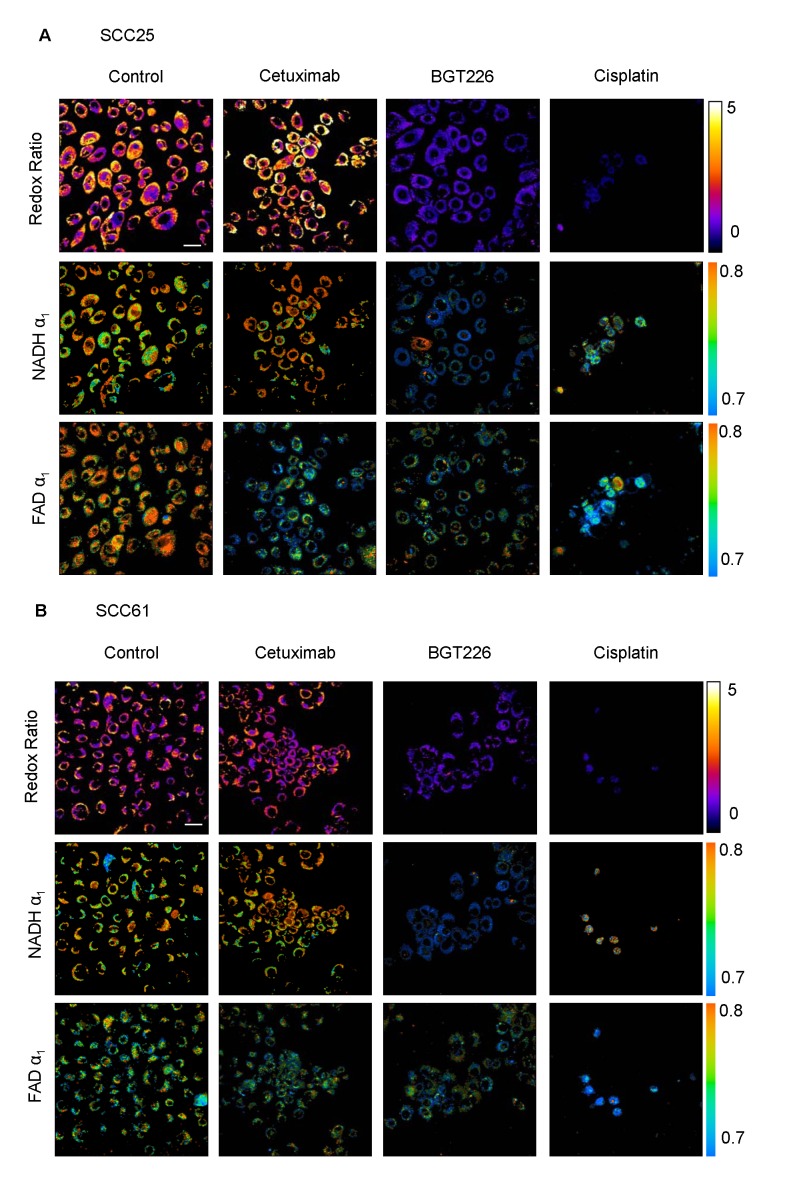
Representative autofluorescence images after treatment. Representative images of the redox ratio (1st row), NADH α_1_ (2nd row), and FAD α_1_ (third row) for (a) SCC25 cells and (b) SCC61 cells treated with control (1st column), cetuximab (2nd column), BGT226 (3rd column), or cisplatin (4th column). α_1_ quantifies the short lifetime component (α_1_+α_2_ = 1). NADH α_1_ represents the contribution from free NADH, while FAD α_1_ conversely represents the contribution from protein-bound FAD. Scale bar represents 30 um.

**Figure 5:**
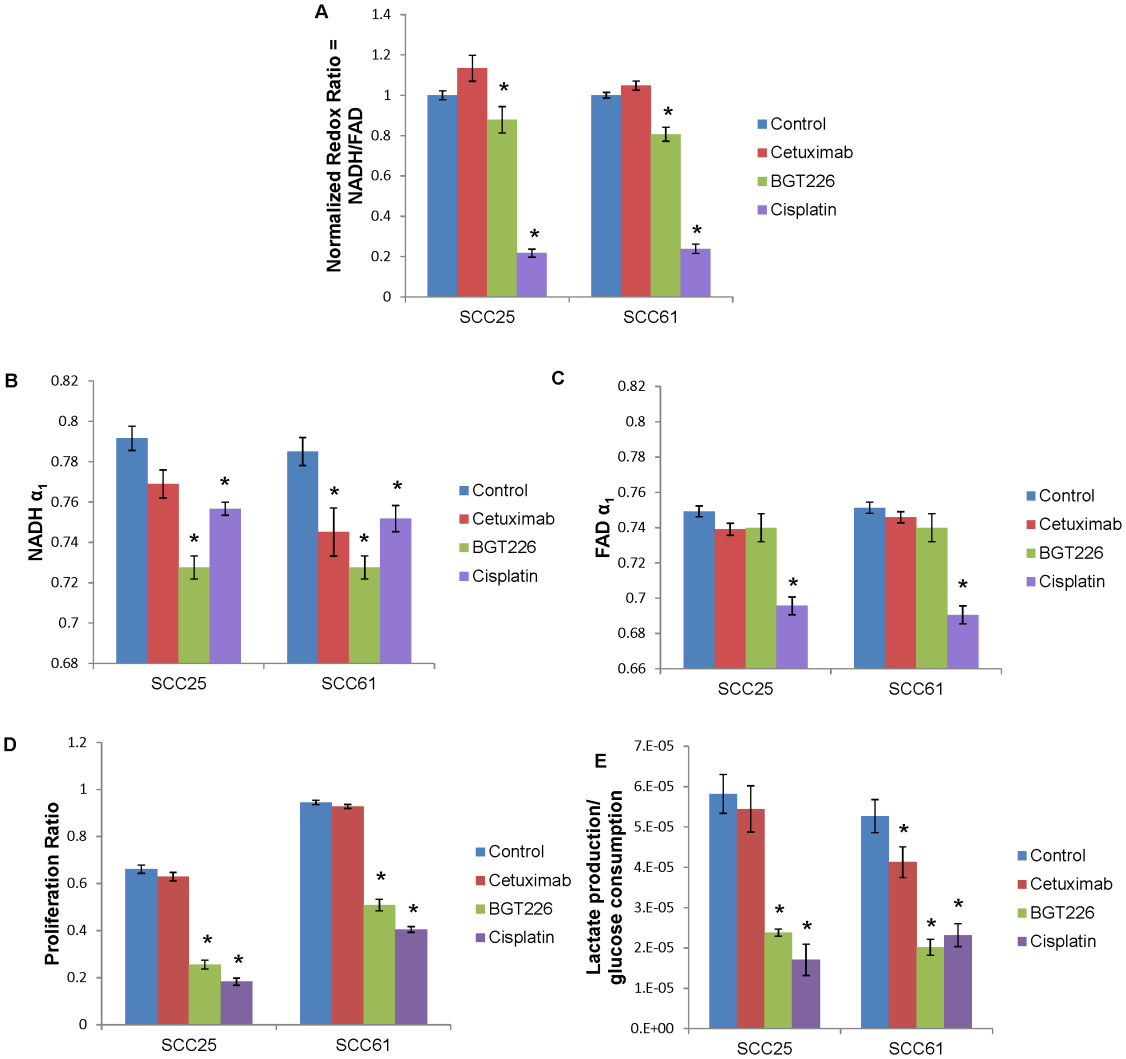
Metabolic endpoints measure response in SCC25 and SCC61 after treatment. (a) SCC25 and SCC61 cells were treated with cetuximab, BGT226, or cisplatin for 24 hours. The optical redox ratio is defined as the fluorescence intensity of NADH divided by that of FAD and is normalized by the redox ratio from control cells per day. Treatment with cetuximab does not affect the normalized redox ratio. Treatment with BGT226 or cisplatin decrease the normalized redox ratio. α_1_ represents the contribution of the short fluorescence lifetime (free conformation for NADH and protein-bound conformation for FAD) (α_1_+α_2_ = 1). (b) NADH α_1_ decreases after treatment with BGT226 and cisplatin in SCC25 cells and after treatment with cetuximab, BGT226, and cisplatin in SCC61 cells. (c) FAD α_1_ decreases after treatment with cisplatin in SCC25 and SCC61 cells. (d) Cells were treated for 24 hours and proliferating cells were labeled with BrdU. The ratio of proliferating cells was calculated by dividing the number of BrdU-labeled cells by the total number of cells per image. Treatment with cetuximab does not affect proliferation. Treatment with BGT226 or cisplatin treatment decrease proliferation. (e) The ratio of lactate production/glucose consumption reflects rates of glycolysis, which decreases after treatment with BGT226 and cisplatin in SCC25 cells and after treatment with cetuximab, BGT226, and cisplatin in SCC61 cells. *p<0.05 rank sum test, compared with control; mean ± SEM.

Representative images of SCC25 and SCC61 cells after 24 hours of treatment provide qualitative visualization of the redox ratio, NADH α_1_, and FAD α_1_ (Figure 4). NADH and FAD fluorescence from the cytoplasm was quantified across treatment groups and cell lines. The redox ratios of SCC25 and SCC61 cells show no significant changes with cetuximab treatment, and decrease with BGT226 and cisplatin treatment (Figure 5a). The fluorescence lifetimes of NADH and FAD reflect cellular microenvironment and protein-binding. NADH α_1_ represents the contribution from free NADH. For SCC25 cells, NADH α_1_ decreases with BGT226 and cisplatin treatment. For SCC61 cells, NADH α_1_ decreases with cetuximab, BGT226, and cisplatin treatment (Figure 5b). FAD α_1_ represents the contribution from protein-bound FAD. For SCC25 and SCC61 cells, FAD α_1_ decreases with cisplatin treatment (Figure 5c). Combined, these data show that optical metabolic endpoints are sensitive to treatment with cetuximab, BGT226, and cisplatin in SCC25 and SCC61.

Proliferation was quantified as a standard measure of treatment response. Cetuximab treatment does not induce a statistically significant effect on proliferation, whereas BGT226 and cisplatin treatment decrease proliferation (Figure 5d). Additionally, glycolytic rates were quantified after treatment. SCC25 shows decreased lactate production/glucose consumption with BGT226 and cisplatin treatment, and SCC61 shows decreased lactate production/glucose consumption with cetuximab, BGT226, and cisplatin treatment (Figure 5e).

For SCC25, the free and protein-bound lifetimes of NADH and FAD (τ_1_ and τ_2_, respectively) show no significant change with any treatment (Table 1a). The NADH mean lifetime (τ_m_) shows no change for any treatment, and the FAD mean lifetime increases with cisplatin treatment (p<0.05). For SCC61, free and protein-bound NADH lifetimes show no significant change with cetuximab and BGT226 treatment and increase with cisplatin treatment (p<0.05, Table 1b). For SCC61, the protein-bound FAD lifetime (τ_1_) shows no change with any treatment, and the free FAD lifetime (τ_2_) increases with cisplatin treatment (p<0.05). The NADH mean lifetime (τ_m_) increases with BGT226 and cisplatin treatment (p<0.05), and the FAD mean lifetime increases with cisplatin treatment (p<0.05). NADH and FAD α_1_ are also listed in Table 1. These data suggest that NADH α_1_ is more sensitive to shifts due to treatment than τ_1_, τ_2_, or τ_m_, and FAD α_1_ is more sensitive to shifts due to treatment than τ_1_ or τ_2_. The NADH and FAD lifetime values for the OKF6 cells are included in Table 1c.

**Table 1:** The short and long fluorescence lifetime components (τ_1_ and τ_2_, respectively), mean lifetime (τ_m_), and contribution of the short lifetime component (α_1_) of NADH and FAD in SCC25 (a) and SCC61 (b) after treatment with cetuximab, BGT226, or cisplatin, as well as in OKF6 (c).

(a)	SCC25				
		Control	Cetuximab	BGT226	Cisplatin
NADH	τ_1_ (ps)	528±29	526±34	468±27	569±37
	τ_2_ (ps)	2899±53	2889±56	2726±43	2955±62
	τ_m_ (ps)	1019±37	1068±39	1083±36	1130±46
	α_1_	0.792±0.006	0.769±0.007	0.728±0.006*	0.757±0.003*
FAD	τ_1_ (ps)	426±24	396±20	372±19	378±18
	τ_2_ (ps)	2667±33	2624±29	2633±24	2630±20
	τ_m_ (ps)	983±30	913±28	959±33	1059±29*
	α_1_	0.749±0.003	0.746±0.003	0.74±0.008	0.696±0.005*
**(b)**	**SCC61**				
		**Control**	**Cetuximab**	**BGT226**	**Cisplatin**
NADH	τ_1_ (ps)	480±26	486±40	460±19	796±54*
	τ_2_ (ps)	2760±43	2763±62	2673±34	3335±60*
	τ_m_ (ps)	968±28	1064±50	1083±36*	1398±50*
	α_1_	0.785±0.007	0.745±0.012*	0.728±0.006*	0.752±0.007*
FAD	τ_1_ (ps)	432±25	363±12	381±20	450±23
	τ_2_ (ps)	2654±35	2546±15	2667±28	2713±25*
	τ_m_ (ps)	978±31	913±17	959±33	1142±30*
	α_1_	0.751±0.003	0.746±0.003	0.74±0.008	0.691±0.005*
**(c)**	**OKF6**				
		**Control**			
NADH	τ_1_ (ps)	551±38			
	τ_2_ (ps)	3035±61			
	τ_m_ (ps)	1209±42			
	α_1_	0.731±0.0039			
FAD	τ_1_ (ps)	455±25			
	τ_2_ (ps)	2706±35			
	τ_m_ (ps)	1037±33			
	α_1_	0.737±0.0043			

*p<0.05 rank sum test, compared with control; mean ± SEM.

## Discussion

Optimized treatment regimens have potential to improve quality of life for HNSCC patients. The goal of this study is to characterize optical metabolic imaging for early assessment of treatment efficacy. The HNSCC cell lines SCC25 and SCC61 were treated with targeted therapies (cetuximab and BGT226) and chemotherapy (cisplatin) for 24 hours, and the optical redox ratio and fluorescence lifetimes of NADH and FAD were quantified. These molecular-level measurements that reflect cellular metabolism could resolve anti-cancer treatment effects sooner than current imaging modalities, including CT, MRI, and PET. Early measurement of treatment efficacy could accelerate drug screening and identify optimal treatment regimens for individual patients, thereby improving patient outcomes.

Isolation of NADH and FAD fluorescence emission was verified using cyanide perturbation (Figure 1). These shifts in the redox ratio and NADH and FAD lifetimes match published results for the MCF10A nonmalignant cell line from the breast [14] [33] [36] [37]. However, these results have been previously unreported in cells from the oral cavity. Optical metabolic imaging distinguishes the malignant SCC25 and SCC61 cell lines from the nonmalignant OKF6 cell line (Figure 2). The increased redox ratio in HNSCC cells reflects increased reliance on glycolysis compared with oxidative phosphorylation, as expected in cancer cells (Warburg effect) [11]. This result agrees with previous findings that the redox ratio reports changes with malignancy [38]. The altered NADH and FAD fluorescence lifetimes reflect distinct signaling pathways in the HNSCC cells compared with nonmalignant cells. HNSCC cells exhibit modified intrinsic metabolic signaling that changes NADH binding sites [39], and the fluorescence lifetimes have been shown to change when NADH or FAD are bound to different enzymes [40]. Previous studies have also shown that fluorescence lifetime imaging distinguishes normal from precancer in the DMBA-treated hamster cheek pouch model [18] [19].

Changes in the redox ratio across treatment groups are consistent with proliferation rates after treatment. The redox ratio is a global measure of cellular metabolism, which drives proliferation. The redox ratio and proliferation ratio are unaffected by cetuximab treatment and show statistical differences with BGT226 and cisplatin treatment (Figure 5). The lack of effect from cetuximab treatment could be attributed to *in vitro* application as a single agent. In addition to inhibiting EGFR as a means of exerting effects, *in vivo* it has been shown that cetuximab initiates antibody-dependent cell-mediated cytotoxicity (ADCC) by binding to EGFR and recruiting natural killer cells and macrophages to digest the targeted cell [41]. However, immune cells are not present in these cell culture studies. *In vivo*, cetuximab treatment would be expected to have a greater impact on the optical redox ratio due to increased cell death through ADCC. This expectation is supported by our previous study, which showed more dramatic changes in redox ratio *in vivo* versus *in vitro* after treatment with the antibody trastuzumab [14]. Additionally, cetuximab is maximally effective in combination with radiotherapy and chemotherapy because it inhibits DNA repair mechanisms [42]. Conversely, BGT226 and cisplatin actively cause autophagy and cell death, respectively, in cell culture [9] [43]. No previous literature has reported the effects of cetuximab or BGT226 on the optical redox ratio. Cisplatin has shown changes in the optical redox ratio in primary human foreskin keratinocytes [44].

The contribution from free NADH (NADH α_1_) shows shifts in protein-binding of NADH with BGT226 and cisplatin treatment in SCC25 and SCC61 cells as well as with cetuximab treatment in SCC61 cells (Figure 5b). The ratio of lactate production divided by glucose consumption reflects amounts of terminal glycolysis compared with total glucose metabolism. In glycolysis, glucose is consumed and pyruvate is produced. Pyruvate is either fermented into lactate as a terminal stage of glycolysis or converted to acetyl-coA as fuel for the citric acid cycle. Cetuximab treatment does not affect glycolysis rates in SCC25, but decreases glycolysis in SCC61. The decrease in glycolysis and NADH α_1_ in SCC61 cells indicates shifts in metabolic pathways in response to treatment. However, proliferation is not affected by cetuximab in SCC61, indicating compensation by effectors downstream of EGFR. Cetuximab has been shown to not affect short-term cell growth in SCC25, which could explain the lack of statistical significance in the NADH α_1_ and glycolytic index [45]. BGT226 and cisplatin treatments decrease glycolysis in SCC25 and SCC61. The effect of cetuximab or BGT226 on SCC61 cells has not been cited in previous literature, and the effect of BGT226 on glycolysis has not been reported in any model. The measurement of glycolysis rates calculated by lactate production/glucose consumption is correlated with NADH α_1_ (0.81 Pearson’s correlation coefficient, p<0.05). No other measurements produced a statistically significant correlation coefficient with lactate production/glucose consumption or proliferation. Cisplatin treatment produces outliers that impacted the correlations, particularly between lactate production/glucose consumption and the redox ratio. Previous studies have shown a correlation between glucose uptake/lactate production and the optical redox ratio in breast cancer cells [14]. However, this correlation was determined for basal metabolic rates in cells without treatment, and cells from a different organ site could rely on different metabolic mechanisms. Additionally, the control SCC61 cells show a higher proliferation ratio than SCC25 (p<0.05) (Figure 5d), but no statistical difference in redox ratio (Figure 2a). This is in contrast to the similar lactate production/glucose consumption between the control SCC25 and SCC61. Although the trends in NADH α_1_ and redox ratio agree with the gold standards, they are not surrogate measurements of proliferation or the amount of glycolysis compared with total glucose metabolism. For example, alternative metabolic pathways such as beta oxidation of fatty acids, the pentose phosphate pathway, and glutaminolysis are all captured differently by these gold standard measurements and our optical measurements [46] [47]. The contribution from protein-bound FAD (FAD α_1_) is unaffected by cetuximab and BGT226 treatments and decreases with cisplatin treatment (Figure 5c).

Early measures of treatment response could enable effective intervention while reducing the acute toxicities and serious morbidities from ineffective therapies. Molecular-level measurements that reflect cellular metabolism are well-suited to measure effects from cancer treatments that target metabolic pathways. The optical redox ratio and fluorescence lifetimes of NADH and FAD resolve a response after 24 hours of treatment with targeted therapies and chemotherapies in HNSCC cells. These results indicate that optical metabolic imaging shows promise to identify effective drug candidates during drug development. Additionally, applying optical metabolic imaging to measure treatment response early has potential to impact quality of life for HNSCC patients.
